# Adult mortality patterns in Yemen before and during armed conflict: evidence from a web survey of the global diaspora

**DOI:** 10.1186/s13031-023-00535-8

**Published:** 2023-08-10

**Authors:** Catherine R. McGowan, Mervat Alhaffar, Promise Ekoriko, Sawsan Al-Refai, Jamal Badr, Lucy Bell, Francesco Checchi

**Affiliations:** 1 Department of Infectious Disease Epidemiology, Faculty of Epidemiology and Population Health, London School of Hygiene and Tropical Medicine, Keppel Street, London, WC1E 7HT UK; 2 Syria Research Group, London School of Hygiene & Tropical Medicine and National University of Singapore School of Public Health, London, UK; 3 Information Technology Services, London School of Hygiene and Tropical Medicine, Keppel Street, London, WC1E 7HT UK; 4 City of Ottawa, Ottawa, K2J 2V4 Canada; 5 Het Grote Midden Oosten Platform [The Greater Middle East Platform], The Hague, Netherlands

**Keywords:** Respondent-driven sampling, Mortality estimation, webRDS, Sibling survival, Child survival, Conflict, Yemen, Diaspora

## Abstract

**Background:**

The ongoing war in Yemen has created a severe and protracted crisis that has left nearly three-quarters of the population in need of urgent humanitarian assistance. Despite eight years of conflict there exist few robust estimates of how the conflict (and the conflict combined with the COVID-19 pandemic) have affected mortality in Yemen. As the security situation has limited access to affected populations we have designed a novel alternative to local mortality surveys.

**Methods:**

We used a web-based, respondent-driven sampling method to disseminate a mortality survey amongst the global Yemeni diaspora. We used Cox proportional hazards survival models to estimate the association between the exposure (i.e. between the pre-conflict, conflict, and conflict/pandemic periods) and mortality risk, adjusted for gender and birth cohort.

**Results:**

Eighty-nine eligible respondents completed the survey. Respondents provided data on the status of 1704 individuals of whom 85 (5%) had died; of these 65 (3.8%) were reported to have died in Yemen. An analysis of survivorship of respondents’ parents after their 50th birthday (adjusted for gender and birth cohort) provided weak evidence that the war and pandemic periods were associated with higher mortality when compared to the pre-war period. Analysis of the subset of individuals who died in Yemen also suggested an increased, but non-significant hazard of dying during the war/pandemic period: this association tended towards significance when allowing for varying degrees of out-migration from Yemen across the cohort. The number of deaths amongst respondents’ siblings and children under five in Yemen were too low to allow meaningful analysis.

**Conclusions:**

Our data suggest increased mortality during the war/pandemic period, compared to the pre-war period, among older Yemeni adults. However, our findings require careful interpretation as our study design cannot establish causation, and as our small and non-representative sample appeared skewed towards higher-income, urban communities. Surveys of diaspora populations offer a promising means of describing mortality patterns in crisis-affected populations; though, large numbers of respondents are likely required to achieve accurate mortality estimates and to adjust for selection bias.

## Background

The ongoing conflict in Yemen has precipitated one of the world’s most severe and protracted humanitarian crises. The COVID-19 pandemic has also compounded existing health and economic challenges. By 2022 nearly three-quarters of the Yemeni population were in need of humanitarian assistance [1]. Accurate measurement of the population-level impacts of the crisis is hampered by disrupted public health surveillance, incomplete vital events registration, and limited access to much of Yemen due to insecurity and bureaucratic impediments [2].

Mortality is a key summative metric for describing the health status of populations [3]. Mortality has additional import as crude measure of crisis-attributable public health impact, and is a most commonly used indicator for assessing the level of humanitarian need in conflict settings [4]. There are several approaches to collecting mortality data in settings lacking a functioning vital registration system. These include retrospective household surveys, prospective community surveillance, key informant interviews, verbal autopsy, body counts, capture-recapture, and statistical models based on available data of known risk factors for mortality [5]. Whilst all methods have inherent limitations (e.g. sampling and response bias for retrospective surveys and verbal autopsies, poor predictive accuracy of regression estimates when risk factor data are incomplete or inaccurate) these are augmented in conflict settings where population displacement and access constraints may render ground surveys infeasible. These obstacles suggest the need for novel, remote methods for measuring population health [6]. With the ongoing crisis in Yemen as the study setting, we have explored three novel methods for estimating mortality in conflict settings using remotely collected data. These methods include: (1) a key-informant study using capture-recapture sampling [7], (2) a satellite imagery analysis of cemeteries [8], and (3) a web-based, respondent-driven sampling (webRDS) survey of the Yemeni diaspora. We report below on our use of the webRDS survey to estimate excess mortality amongst older adults in Yemen due to the ongoing conflict and the COVID-19 pandemic.

## Methods

### Study design

We designed a web-based mortality survey to collect information from members of the global Yemeni diaspora about the status of their close family members in Yemen. The survey included questions about the: (1) location (i.e. governorate), age at time of death, and manner of death of deceased family members living in Yemen (including parents, biological siblings, and nieces/nephews of the respondent and of the respondent's current or most recent living or deceased spouses), and (2) the age (at the time of survey completion) of all living family members. The survey also asked respondents to provide proxy indicators- based on the questionnaire used in the most recent Yemen National Health and Demographic Survey (HDS) - of the socio-economic status (SES) of their Yemeni-based family members [9]. Respondents were eligible to complete the survey if they resided outside of Yemen and were aged 18–49. We restricted our study sample to diaspora populations to assess the extent to which diaspora populations are willing and able to provide information about deaths in Yemen, and because formative interviews and focus groups with diaspora networks, conducted ahead of the study, suggested that resident Yemenis would be reluctant to provide data on conflict-related mortality due to concerns about security and confidentiality.

### Sampling approach

We opted to use a respondent-driven sampling (RDS) approach to recruitment [10]. RDS relies on chains of peer-referral rather than recruitment directly by the study team with respondents recruiting other eligible respondents from within their networks. This method has been traditionally been used to recruit hard to reach populations, or those with poorly understood sampling frames, including alcohol and drug users [11], men who have sex with men [12, 13], and workers with precarious employment [14]. RDS has traditionally involved face-to-face recruitment; however, RDS surveys have also been carried out using digital platforms (a.k.a. webRDS). As there was no commercially available webRDS solution we developed our own; the solution is comprised of a survey platform (i.e. ODK), and a bespoke webRDS system. A detailed description of our use of RDS, as well as the development (and limitations) of the webRDS solution, has been published elsewhere [15].

The RDS approach requires initial recruitment of *seeds* (i.e. individuals with social networks that are likely to include significant numbers of eligible respondents) within the study population. We were able to identify *seed* respondents through our existing networks within the Yemeni diaspora, established during scoping work for our other mortality estimation studies. We sent a link to the survey to *seed* respondents via our webRDS administrator interface. *Seeds *were asked to complete the survey and then send it to five potential respondents within their social network(s). Onward survey invitations were managed by respondents without interference from the study team. Each downstream respondent was asked to further cascade the survey to five other potential respondents via the webRDS user interface. Respondents were asked to complete the survey for themselves (in English or Arabic) and, if possible, on behalf of a current/former spouse.

### Sample size

We used a simulation to compute a target sample size that would enable us to observe an association between the crisis (war and pandemic) period and <5 mortality (deaths per live births before age five years) with power ≥ 80% and 5% significance (see https://github.com/francescochecchi/yem_diaspora_mortality). Specifically, we constructed varyingly large samples of diaspora respondents with assumed attrition 20% and 50% of siblings still living inside Yemen, and a mean 6.0 (standard deviation = 1.0) siblings per respondent based on average fertility 30 years prior to the survey. Based on the most recent UN estimates, we further assumed: pre-crisis neonatal (first month), post-neonatal infant (months 1 to 11) and child (months 12 to 59) mortality rates per 1000 live births of 43, 27 and four respectively (adjusted downwards by one third assuming selection bias of respondents towards higher-income, healthier families); varying relative risks of death during the crisis period, compared to pre-crisis; and an annual crude birth rate of 48 per 1000. After allowing this population to reach equilibrium, we tracked total deaths before 59 months of age in the 48 months before and after the start of the crisis, and computed the one-sided p-value for the difference between mortality during the crisis and non-crisis periods. After 100 simulations, we estimated that around 1000 survey respondents would be required to observe a relative <5 mortality increase of 40%.

### Analysis

As this study involved novel methods we were prepared to adapt our approach to analysing the data based on the features of the data we were able to obtain. There is an established approach to analysing RDS surveys which assumes randomness in each respondent’s onward recruitment and weights each observation to minimise selection bias [10]. However, this approach requires a large independent sample of peer-recruited respondents (comprised of the combined network respondents of each *seed*). As our sample included proportionately few peer-recruited respondents—18 [20%] of the total 89 respondents—RDS analysis would not have been appropriate; thus, we treated our sample as a set of uncorrelated responses with equal selection probabilities (see Discussion).

We used data from the mortality survey to produce sub-group survival estimates for older adults (50 + years). Due to the low numbers of deaths amongst siblings and children <5—there were only three deaths in children <5 during the period of analysis—we present only mortality analysis for ‘older adults’. We defined older adults as those aged 50 years or greater and included in this subset observations representing parents of respondents, or the parents of their spouse (if provided). After exploring patterns in survival based on different factors (including gender and years of birth (i.e. birth cohort, age)) using Kaplan–Meier survival curves, we fitted Cox proportional hazards survival models to estimate the association between period—i.e. pre-conflict (pre-June 2014) as the baseline, conflict (June 2014 to March 2020), and pandemic (April 2020 onwards) as the exposures—and mortality risk from age 50 onwards (considering all deaths, and only deaths within Yemen), adjusted for gender and birth cohort. As we wished to quantify the effect of the exposure on survival at different ages, we used age itself as the model’s time metric. We split each individual’s time trajectory into age segments bounded by their 50^th^ birthday, the age (after 50 years) at which they entered the conflict and pandemic periods, the age at which they exited each period (through death or passage of time), and their age on the date the survey was completed. Each segment was attributed a survival outcome based on whether death occurred within it. We omitted any segments, or portions thereof, in which the individual was no longer alive or had not yet turned 50 years old. Individuals’ ‘crisis’ and ‘pandemic’ segments were considered exposures, with the ‘pre-crisis’ segment as the reference or baseline.

As we did not ask whether and when individuals in the cohort migrated out of Yemen, it is plausible that the survival hazard ratios would have been biased by over-estimated exposure time, since we assume in our baseline analysis that no one in the cohort migrated out. To relax this assumption, we did sensitivity analysis in which, over many random simulations, we attributed to each individual who did not die in Yemen a varying binomial probability of having migrated out and, for individuals selected in the simulation as having migrated, a varying fraction of their analysis period spent within Yemen before migration. We did 100 simulations for each combination of migration probabilities and fractions of the period spent in Yemen. We analysed the data using R version 4.2.0 (2022-04-22) [16].

### Ethics

Information about *seed* respondents was limited to an email address or phone number [15]. Onward respondents were invited using an email address or phone number inputted by the inviting respondent.  We collected no personal, or otherwise potentially identifying information from respondents; the survey did not include free text fields. Potential respondents were required to confirm eligibility and indicate their consent to participate in the study within the survey itself. The study was reviewed and approved by the London School of Hygiene & Tropical Medicine (LSHTM) Observational Research Ethics Committee (REC# 25672).

## Results

Eighty-nine respondents met the study eligibility criteria (of the 93 who completed the survey between 3 March and 15 August 2022) constituting a sample considerably below the target sample size (see: McGowan et al. [15] for possible reasons). Reasons for ineligibility included: being outside the eligible age range (i.e. 18–49) [n = 3] and having a close family member who had already completed the survey [n = 1]. Respondents were only asked to provide information about their close family members (referred to as *individuals* or, collectively, *study population* hereafter) and those of their spouse and, as such, were not themselves included as observations. The only information we requested about the respondents was their location of birth (Table 1), estimated network size, and marital status.

**Table 1:** Distribution of respondents by place of birth, per million population

Governorate	Estimated population size (see: Checchi et al. 2022)	Number of respondents	Respondents per million population
Abyan	703,000	1	1.4
Ad Dali'	759,000	0	0
Aden	943,000	7	7.4
Al Bayda	907,000	2	2.2
Al Hodeidah	3,270,000	1	0.3
Al Jawf	680,000	0	0
Al Maharah	148,000	0	0
Al Mahwit	807,000	0	0
Amran	1,501,000	0	0
Dhamar	2,188,000	0	0
Hadramawt	1,568,000	1	0.6
Hajjah	2,273,000	0	0
Ibb	3,412,000	2	0.6
Lahj	1,148,000	0	0
Ma'rib	668,000	0	0
Raymah	629,000	0	0
Sa'dah	964,000	0	0
Sana'a	1,331,000	31	23.3
Sana'a City	2,723,000	13	4.8
Shabwah	762,000	0	0
Socotra	70,000	0	0
Ta'iz	3,699,000	8	2.2
[not born in Yemen]		23 (26%)	
Total	31,153,000	89	

Only a quarter (26%) of respondents were born outside of Yemen. Of the 66 respondents born in Yemen nearly 80% were born in Sana’a (n = 31, 47%), Sana’a City (n = 13, 20%), or Ta’iz governorates (n = 8, 12%). Of the 87 respondents who provided information about marital status, 30 (34%) had never been married, and 57 (66%) indicated that they were currently or had been married. Respondents who reported having a spouse of Yemeni origin (n = 53) were asked to also complete the survey on their behalf. In total we collected mortality data from 89 respondents, and 53 spouses of respondents, for a total of 142. Overall, respondents provided information about 1704 individuals of which 284 were parents (106 of which were parents of spouses), 604 were siblings (197 of which were siblings of spouses), and 816 were nieces/nephews (301 of which were nieces/nephews of spouses).

### Characteristics of the study population

Respondents were asked to provide information about the SES of their own close family members still residing in Yemen; respondents were not asked to provide SES information about their spouse’s family. Proxy indicators of SES compared to the 2013 HDS for Yemen are presented in Table 2 [9]. Comparing proxy SES indicators for family members described as ‘urban’ to those of the urban 2013 HDS population suggests higher overall welfare and greater socioeconomic condition (as indicated by asset ownership) amongst Yemen-based family members of our study respondents. However, it is notable that some housing characteristics (e.g. primary water source) suggest otherwise. The number of respondents who provided proxy SES indicators for ‘rural/unknown’ Yemen-based family members was small (n = 9); however, these data also suggest slightly higher household wealth amongst our study population. As the most recent Yemen HDS is from 2013, we cannot account for changes in population demographics within Yemen and are thus unable to determine how our study population compares to the current Yemeni population.

**Table 2:** Indicators of socio-economic status amongst respondents’ closest family members living in Yemen compared to the 2013 Yemen National Health and Demographic Survey

	Number (%) of respondents		Yemen HDS 2013	
	Urban (*n* = 80)	Rural/Unknown (n = 9)	Urban (de jure) (%)	Rural (de jure) (%)
**Asset ownership**				
Air conditioner	23 (28.75%)	1 (11.11%)	**28.4**	**4.9**
Fan	38 (47.5%)	5 (55.56%)	*49.2*	**13.7**
Generator	61 (76.25%)	5 (11.11%)	**23.2**	*13.9*
Mobile telephone	79 (98.75%)	9 (100%)	**93.5**	**74.0**
Landline telephone	58 (72.5%)	4 (44.4%)	**37.0**	**7.8**
Radio	49 (61.25%)	7 (77.78%)	**41.2**	**39.4**
Refrigerator	77 (96.25%)	5 (11.11%)	**77.3**	*22.7*
Television	78 (97.5%)	7 (77.78%)	**93.9**	**54.5**
Washer	73 (91.25)	2 (22.22%)	**74.7**	**17.6**
Water heater	54 (67.5%)	9 (100%)	**31.6**	**6.4**
**Flooring**				
Cement	13 (16.25%)	4 (44.4%)	*37.8*	**44.1**
Dirt or clay	–	4 (44.4%)	*6.2*	*44.9*
Marble	10 (12.5%)	–	**10.3**	*1.0*
Plaster	1 (1.25%)	–	*1.7*	*2.6*
Stone	2 (2.5%)	1 (11.11%)	**2.3**	**1.8**
Tile	54 (67.5%)	2 (22.22%)	**41.6**	**5.4**
**Type of excreta disposal**				
Improved: single-household sewage pipe	52 (65%)	4 (44.4%)	**61.4**	**5.4**
Improved: single-household septic tank	11 (13.75%)	1 (11.11%)	*21.5*	*25.1*
Improved: shared sewage pipe	7 (8.75%)	2 (22.22%)	**3.1**	**0.7**
Improved: shared septic tank	2 (2.5%)	1 (11.11%)	**1.2**	**3.1**
Unimproved: bucket latrine	1 (1.25%)	–	*2.3*	*9.8*
Unimproved: pit latrine	7 (8.75%)	1 (11.11%)	**7.1**	*20.3*
**Primary water source**				
Improved: bottled	24 (30%)	–	*31.5*	*0.9*
Improved: piped (government)	26 (32.5%)	–	*40.1*	*10.8*
Improved: piped (local agency)	9 (11.25%)	2 (22.22%)	**1.8**	**13.9**
Improved: rainwater collection	–	2 (22.22%)	*0.3*	**4.5**
Improved: protected well or borehole	1 (1.25%)	2 (22.22%)	*2.3*	**20.0**
Unimproved: spring	1 (1.25%)	–	**0.3**	*13.9*
Unimproved: tanker truck	15 (18.75%)	1 (11.11%)	*22.3*	**10.4**
Unimproved: unprotected well	1 (1.25%)	2 (22.22%)	**0.5**	**18.0**
Unimproved: other	3 (3.75%)	–	**0.7**	*7.6*
**Means of transport ownership**				
Animal-drawn cart	1 (1.25%)	2 (22.22%)	**0.7**	**1.6**
Bicycle	1 (1.25%)	–	*18.1*	*7.0*
Boat with motor	1 (1.25%)	1 (11.11%)	**0.7**	**0.8**
Motorcycle or scooter	7 (8.75%)	2 (22.22%)	*13.2*	**12.4**
Car	74 (92.5%)	9 (100%)	28.5	17.4
Minibus	32 (40%)	–		
Truck	2 (2.5%)	–		

Numbers in bold are lower than those describing our sample; numbers in italics are higher

**Fig. 1:**
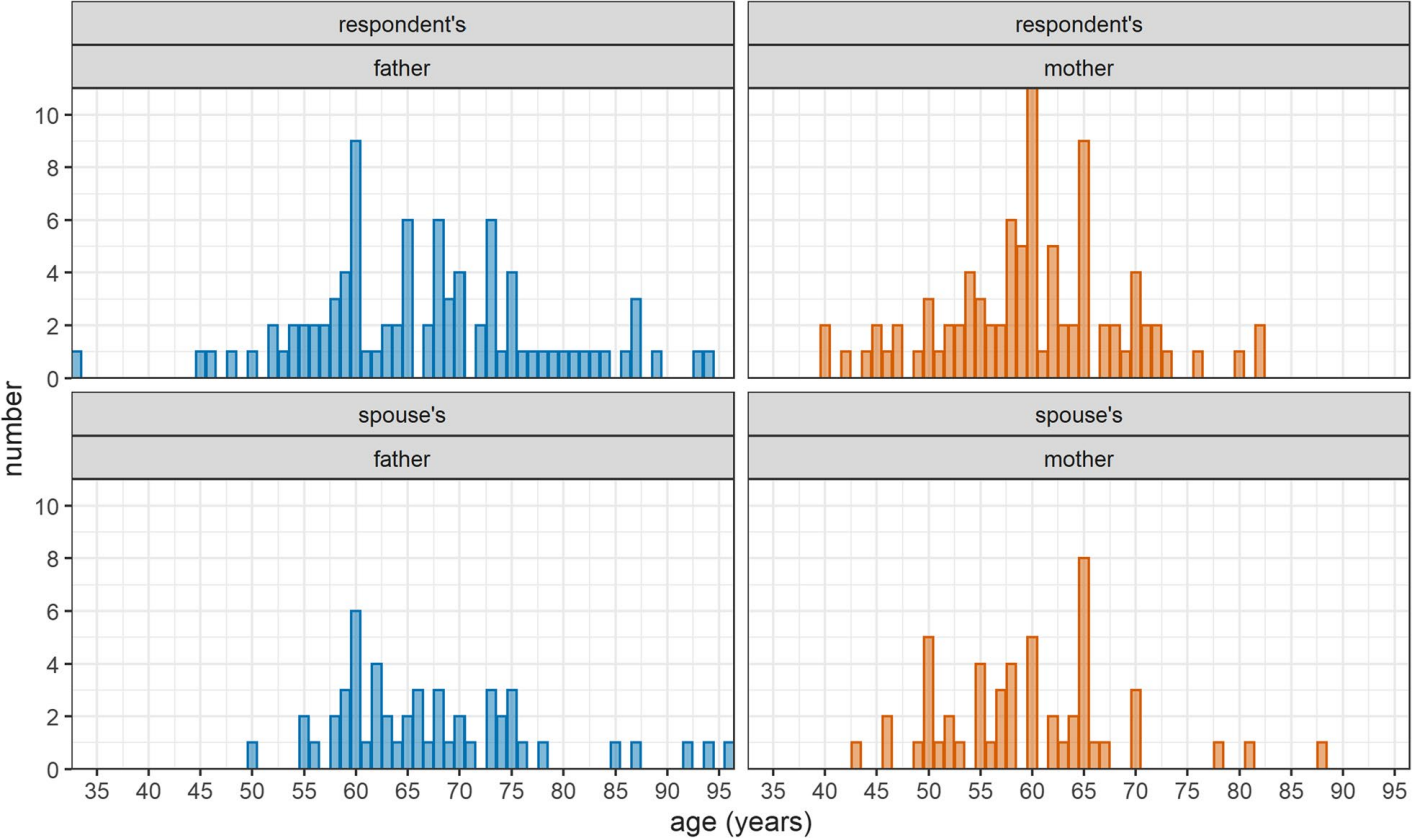
Age distribution of respondents’ and spouses’ parents, by parent’s gender [*Note*: the theoretical age of parents who died outside of Yemen (n = 15) is calculated as of the date of the completion of the survey]

**Fig. 2:**
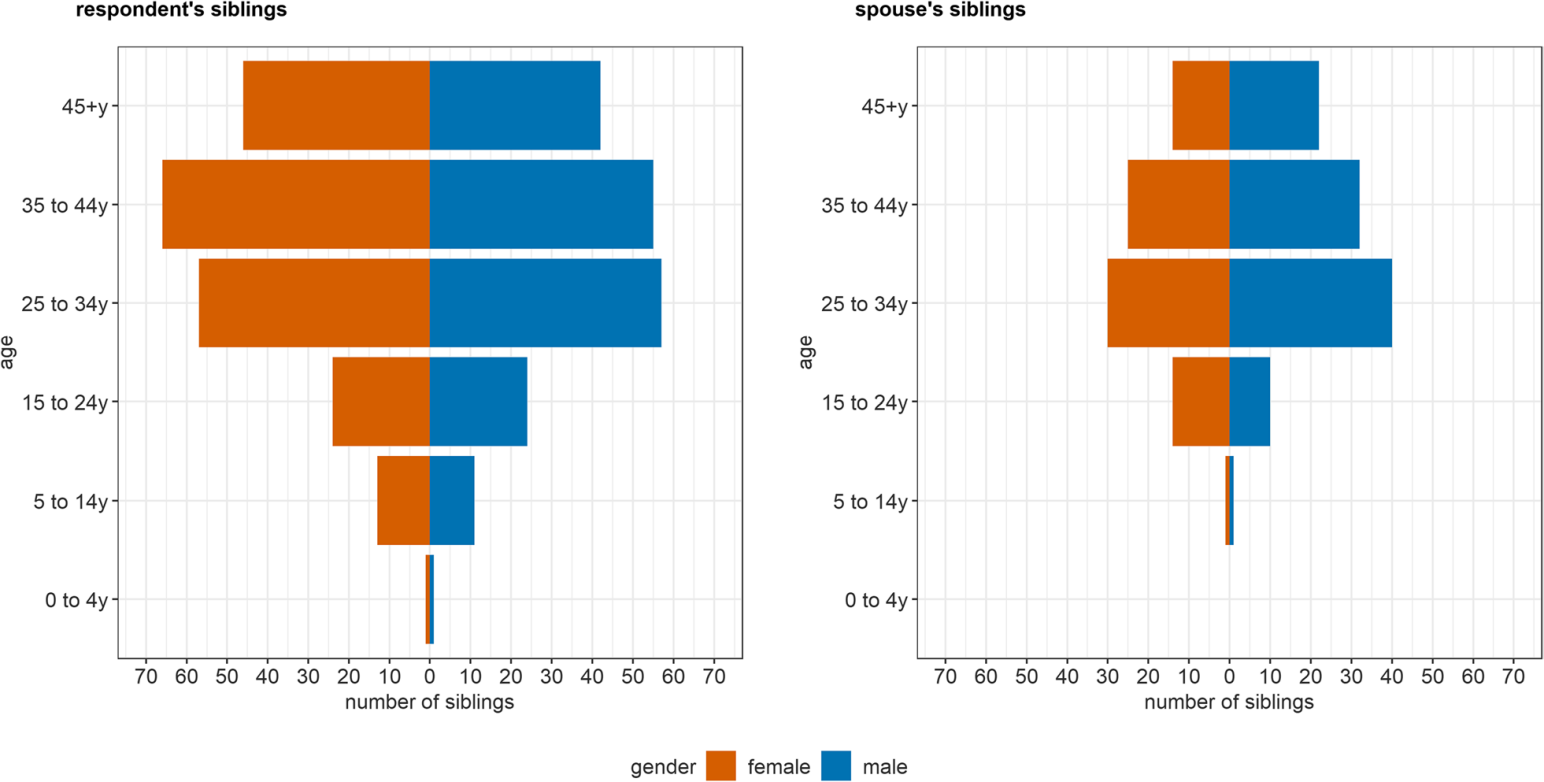
Age and gender distribution of respondents’ and spouses’ reported biological siblings

The mean age was 67 years for fathers (68 years for spouses’ fathers), and 60 years for mothers and spouses’ mothers. There was visual evidence of digit preference/age heaping (e.g. respondents who were unsure about the age of parents may have rounded up or down to the nearest five or ten) amongst parents. The age distribution of parents is presented in Fig. 1. Amongst respondents’ siblings we observed an even gender distribution across age categories; however, amongst spouses’ siblings there appeared to be a preponderance of males (Fig. 2).

**Fig. 3:**
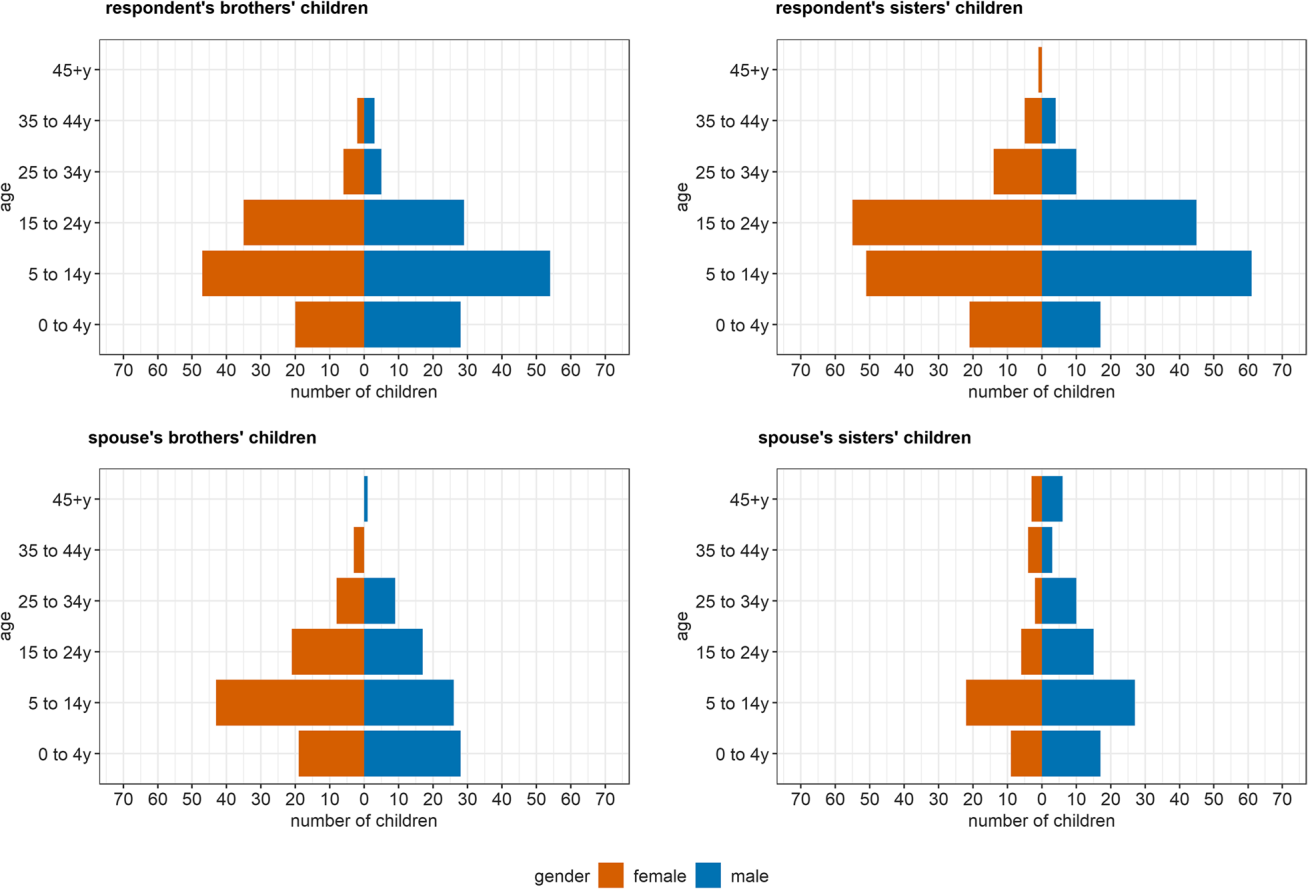
Age and gender distribution of respondents’ and spouses’ reported nieces or nephews, by gender of the parent

The mean age of siblings was consistent across groups: 35 years for brothers, sisters, and spouses’ sisters; and 36 years for spouses’ brothers. The gender distribution amongst nieces/nephews was largely balanced across age groups; however, spouses’ brothers had more children than spouses’ sisters (Fig. 3).

When considering only the respondent’s female siblings and their biological children—likely the least biased of parent-children samples—the total fertility rate (defined as the mean number of children per living female sibling aged 15–49 years) was 2.4 (compared to 4.4 as reported in the Yemen HDS [9]).

### Mortality patterns

Of the 1704 individuals in the study population the majority were reported to be alive (n = 1591, 93.4%). Only 85 individuals were reported to have died (5%), with most deaths occurring amongst parents or spouses’ parents (n = 64, 75% of  deaths) (Table 3).

**Table 3:** Vital status of individuals in the sample, by familial relationship

Relationship	Alive	Deceased	Unknown	Total
Parents of respondent or spouse	214 (75.3%)	64 (22.5%)	6 (2.1%)	284
Siblings of respondent or spouse	574 (95.0%)	12 (2.0%)	18 (3.0%)	604
Nieces/nephews of respondent or spouse	803 (98.4%)	9 (1.1%)	4 (0.5%)	816
All individuals	1591 (93.4%)	85 (5.0%)	28 (1.6%)	1704

Of the 85 individuals reported to have died, 65 (76%) died in Yemen (29 of whom died before 2008). Deaths stratified by period of analysis are presented in Table 4.

**Table 4:** Period and location of reported deaths, by familial relationship

	Pre-analysis period (Pre-January 2008)				Pre-war period (January 2008–May 2014)				War period (June 2014–march 2020)				War and pandemic period (April 2020–August 2022)			
	2008				2014				2020				2022			
	Parents	Siblings	Nieces/nephews	Total	Parents	Siblings	Nieces/nephews	Total	Parents	Siblings	Nieces/nephews	Total	Parents	Siblings	Nieces/nephews	Total
Died outside of Yemen	1	1	0	2	4	1	0	5	5	1	2	8	5	0	0	5
Died in Yemen	23	4	2	29	8	2	1	11	14	2	3	19	4	1	1	6
Total	24	5	2	31	12	3	1	16	19	3	5	27	9	1	1	11

The cause of death amongst the 65 individuals who died in Yemen was reported as disease (n = 29, 44.6%), intentional injury (n = 5, 7.7%), accidental injury (n = 2, 3.1%), and *other* or *unknown* causes (n = 29, 44.6%), with no discernible patterns over time (data not shown).

**Fig. 4:**
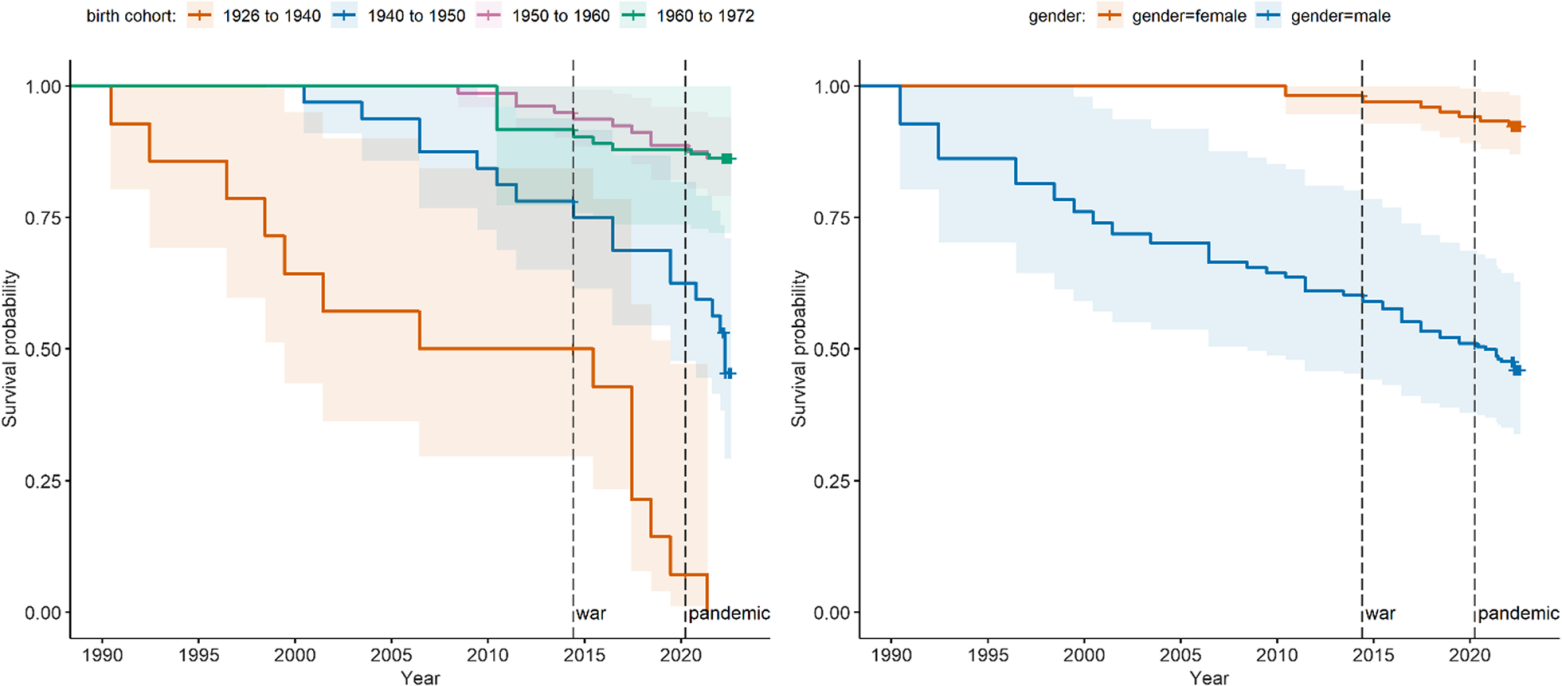
Unadjusted Kaplan–Meier probability of survival in older adults by calendar year, by birth cohort (left panel) and gender (right panel) [Shaded areas indicate 95% confidence intervals]

There was some graphical evidence of an acceleration of mortality after the start of the war period (June 2014) amongst parents and spouse’s parents in older age cohorts. However, our estimates lack precision due to the wide confidence intervals (Fig. 4). When considering all deaths (including those that occurred outside of Yemen), and after adjusting for birth cohort and gender, we found weak evidence that the war and pandemic periods, or the war period and the pandemic period combined, were associated with a two to threefold increase in hazard of dying compared to the pre-war period (Table 5).

**Table 5:** Cox proportional hazards model estimates of the association between period (war and/or pandemic) and survival beyond age 50y

Model (Number of individuals at risk, number of mortality events)			Period	Adjusted† hazard ratio (95% confidence interval)	*p*-value for association	*p*-value for proportional hazards assumption‡	*p*-value for likelihood ratio comparing model to null
1	Outcome: death anywhere Exposure: war or pandemic periods	(N = 246, n = 47)	Pre-war	1.00 [baseline]	–	0.353	0.012
			War	2.48 (0.98 to 6.30)	0.055		
			Pandemic	3.63 (1.13 to 11.70)	0.030		
2	Outcome: death of Yemen resident Exposure: war or pandemic periods	(N = 246, n = 35)	Pre-war	1.00 [baseline]	–	0.211	0.042
			War	1.97 (0.71 to 5.47)	0.191		
			Pandemic	1.80 (0.43 to 7.62)	0.427		
3	Outcome: death anywhere Exposure: war + pandemic period	(N = 246, n = 47)	Pre-war	1.00 [baseline]	–	0.128	0.008
			War/pandemic	2.63 (1.07 to 6.51)	0.036		
4	Outcome: death of Yemen resident Exposure: war + pandemic period	(N = 246, n = 35)	Pre-war	1.00 [baseline]	–	0.161	0.023
			War/pandemic	1.95 (0.71 to 5.34)	0.195		

^†^All estimates of the exposure-outcome association are adjusted for birth cohort and gender

^‡^A *p*-value < 0.05 indicates that the proportional hazards assumption is violated

**Fig. 5:**
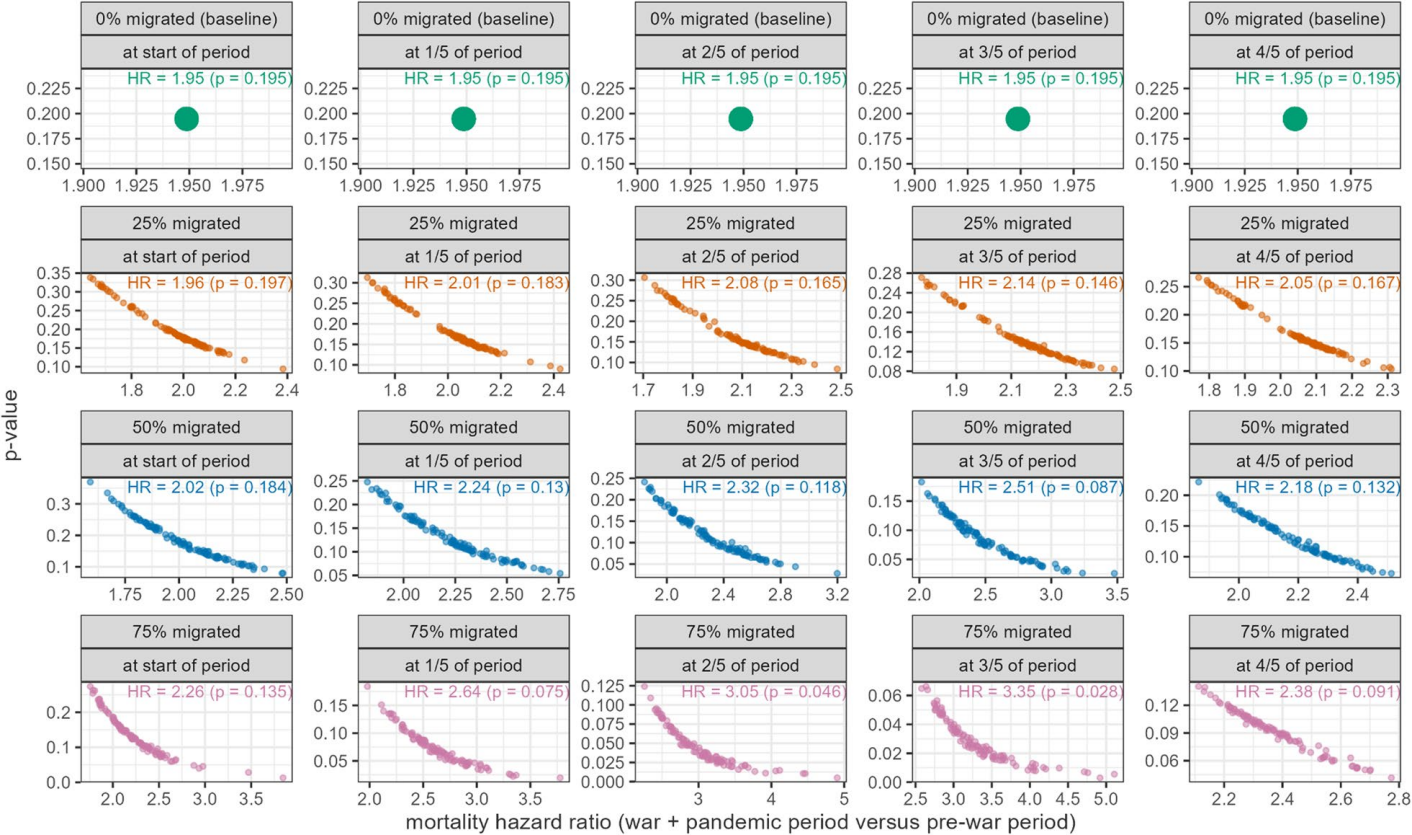
Sensitivity analysis to explore varying proportions of people migrating out of Yemen and the relative point in their period at risk when they left [Each panel shows a sensitivity scenario and each point is a single simulation. The mean hazard ratio (HR) and *p*-value arising from all simulations within a scenario is shown in each panel]

When the sample was restricted to only those individuals who died in Yemen, these associations had a similar directionality but lower magnitude and were nonsignificant. As shown in Fig. 4, hazard ratios tended to increase and become more significant if the analysis was corrected for varying percentages of people leaving Yemen, and fractions of the period when these departures occurred. The most significant associations were predicted if a majority of the cohort did in fact migrate out of Yemen, doing so at around the mid-point of the analysis period, which would have meant a smaller exposure person-time during the war and pandemic periods (Fig. 5).

## Discussion

We found very weak evidence that, within a mostly urban and plausibly higher-income sample of Yemeni older adults, mortality may have increased considerably since the onset of countrywide armed conflict in 2014. Amongst younger ages, data were too sparse to support meaningful inference. To our knowledge this is the first instance of health status within a country being measured through a survey of its diaspora and contributes evidence towards a better quantitative understanding of the impacts of the crisis on Yemenis’ health.

Our findings broadly corroborate results of two other studies of mortality in Yemen that our group has conducted: a key informant study complemented by capture-recapture analysis found two- to ten-fold adult death rate elevations from the plausible pre-conflict baseline, whilst an analysis of cemetery satellite imagery suggests that burial rate was approximately double the non-crisis counterfactual [7, 8]. While these studies have mostly non-overlapping samples and periods, they paint a consistent picture of excess mortality. Our diaspora survey design, whilst prone to selection and recall bias, is based on standard child, sibling, and parent demographic questionnaires and thereby enables mortality data collection from much further back in time than the above methods, or more commonly used retrospective surveys, though in this instance of the survey’s implementation the much lower-than-expected sample size impeded most analysis.

A recent study based on Standardized Monitoring and Assessment of Relief and Transitions (SMART) survey data from 2015 to 2019 concluded that the crude mortality rate (CMR) in Yemen was 0.20 (95% CI 0.17–0.24) per 10, 000 population during the conflict period, compared to 0.19 (95% CI 0.17–0.22) per 10, 000 at baseline [17]. Projecting this rate difference to the population the researchers estimated that there were 168, 212 excess deaths during the five year conflict period, representing a 17.8% increase in overall deaths from baseline. However, an acknowledged limitation of this work was underrepresentation from insecure, hard-to-access areas (the main constraint we sought to address using remote data collection methods). By comparison, a 2020 cluster sample survey in Northwest Syria estimated that the CMR increased 3.55 times (from 16.07 [95% CI 9.91–22.23] to 57.12 [95% CI 48.95–65.30] per 10, 000, *p* < 0.001) annually during the 2011–2020 conflict period, when compared to the pre-conflict period [18].

A cluster sample survey carried out in Iraq calculated a relative risk of all-cause mortality of 2.5 (95% CI 1.6–4.2) when comparing the pre-invasion (5.0 per 1000 people per year [95% CI 3.7–6.3]) to post-invasion (12.3 [95% CI 1.4–23.3]) periods amongst all age groups [19]. Another study of mortality in Iraq reported a pre-invasion crude mortality rate of 2.89 (95% CI 1.56–4.04) per 1000 person years compared to 4.55 (95% CI 3.74–5.27) for the post invasion period [20]. A further study reported higher estimates than Roberts et al. [19, 20], concluding that all cause mortality had increased from 5.5 (95% CI 4.2–7.1) to 13.3 (95% CI 10.9–16.1) deaths per 1000 people per year [21]. Another household survey study estimating violence-related deaths in Iraq between 2002 and 2006 observed an increase in volent deaths from 10.5% (pre-invasion) to 23.2% (post-invasion); and an increase in all cause mortality rates per 1000 person-years from 3.17 (95% CI 2.70–3.75) to 6.01 (95% CI 5.49–6.60) [22]. However, security concerns limited access to high-violence household clusters; thus statistical imputation was used to create estimates for missing households [22].

Though these estimates of excess mortality from crisis-affected countries in the Middle East differ, they broadly suggest increases in mortality in the region of the hazard ratio (2.63, 95% CI 1.07–6.51) from our sub-sample of older adults in Yemen.

### Limitations

Our results should be interpreted with caution. The small sample size, coupled with the small number of deaths in our sample (particularly within the sub-sample of those who died in Yemen), limited the precision of our estimates. In addition, our study design does not allow us to establish causality; as such, we cannot demonstrate that the observed increase in mortality is a consequence of the conflict in Yemen. Another important consideration is that age heaping may have distorted the age distribution in our sample and may have influenced our survival analysis of older adults. As we are unsure if respondents were mainly ‘rounding-up’ or ‘rounding-down’ it is difficult to account for this effect in our analysis. As the number of deaths in our sample is small we cannot readily assess heaping amongst these individuals; however, other studies have observed that heaping is amplified for decedents compared to those who are still alive [23]. Our small sample also limited the degree to which we were able to meaningfully disaggregate our data (e.g. by governorate) and thus add nuance to our findings.

Our survey did not include questions about the residence history of family members, which has likely resulted in some over-estimation within our survival analysis of person-time spent within Yemen (and thus time at risk). Sensitivity analysis suggests that, had we collected information on migration events and dates, a higher hazard ratio would have been estimated. Such data should be collected if possible in future use of this method. It is worth noting that we had originally planned to analyse our data using a statistical model specific to RDS studies and had designed the survey to support this model. We initially opted to take an RDS approach as it is suitable both for recruiting typically hard to reach populations, and for overcoming the limitations of other chain-referral methods by generating a probability sample from an initially non-random sample [10]. As the survey did not cascade to the extent required by the RDS model, we treated the sample as arising from a simple random sampling survey process, which ignores correlation among responses within a referral chain (however, these were a minority of the total respondents as only 18 [20%] of the 89 respondents had been invited by another respondent). It is possible that failure to account for such correlation may have biased association estimates or resulted in overly narrow confidence intervals.

In addition, we are not able to determine the representativeness of our data. Though there appear to be some potentially meaningful differences between our study population and the population in Yemen—particularly with respect to household wealth and geographic representation—we are unable to determine to what extent our findings are generalisable given that the most recent Yemen population data are from 2013. As increased household wealth is likely protective—insofar as it decreases conflict-related risk of death (e.g. by increasing tolerance of food insecurity and health shocks, or increasing mobility and opportunities to migrate)—we may have overestimated survivorship (amongst older adults). Furthermore, we are aware that some respondents started but did not complete the survey. We suspect that those with large families may have been more likely to abandon the survey due to the increased time-investment required to answer questions about many family members. Informal feedback relayed to us via *seed* respondents suggested that some respondents who found the survey distressing thus chosing not to complete it and/or send it to others who might find it similarly distressing. We suspect this may represent a bias in favour of families who have not experienced recent, or particularly traumatic bereavement.

Life-history surveys are typically subject to recall bias with recall tending to worsen with length of recall [24]. In the context of our study this may have inflated the number of more recent deaths. Our study also relies on the respondent’s knowledge of the reproductive history of their close family members which, in some cases, may not be complete or accurate.

Finally, vital status was classified as ‘unknown’ for 28 (compared to 85 individuals reported to be deceased) of the 1704 individuals included in the survey; we cannot discount the possibility that deaths are overrepresented in this group, downward-biasing our association estimates.

## Conclusions

Our findings suggest a decreased probability of survival amongst older adults during the war period when compared to the pre-war period. Despite numerous limitations, we believe that in settings with limited ground access, collecting mortality data from the diaspora is an efficient, anonymous, and potentially informative option that should be documented further. Future studies employing this method will need to develop strategies to increase study participation not only to improve overall precision, but also to allow for age/gender/location stratification. Though methods have been proposed to reduce recall bias and age heaping in sibling studies it is not clear to what extent these could be incorporated into an online survey [25]. We recommend further testing of the webRDS approach alongside improved steps to overcome the various limitations we have identified, and incentivise participation by diaspora members.

## Data Availability

The dataset supporting the conclusions can be downloaded from GitHub [https://github.com/francescochecchi/yem_diaspora_mortality]. The source code for the RDS Solution can be downloaded from Bitbucket [https://bitbucket.org/lshtm-public/rds-api/src/master/; https://bitbucket.org/lshtm-public/rds-ui/src/master/].

## Abbreviations

CMR: Crude mortality rate
HDS: Health and demographic survey
LSHTM: London School of Hygiene and Tropical Medicine
RDS: Respondent-driven sampling
SES: Socio-economic status
SMART: Standardized monitoring and assessment of relief and transitions
webRDS: Web-based respondent-driven sampling
